# Hybrid fluoroscopy–neuronavigation technique for percutaneous balloon compression in trigeminal neuralgia: how I do it

**DOI:** 10.1007/s00701-026-06856-w

**Published:** 2026-04-14

**Authors:** Santi Samuele, Luglietto Davide, Montano Nicola, Ricciuti Riccardo Antonio

**Affiliations:** 1 Department of Neurosurgery, Università Cattolica del Sacro Cuore, Rome, Italy; 2 Department of Neurosurgery, San Camillo Hospital, Rome, Italy

**Keywords:** Trigeminal neuralgia, Percutaneous balloon compression, Neuronavigation, Fluoroscopy, Chronic pain

## Abstract

Percutaneous balloon compression is an established treatment for trigeminal neuralgia, especially in patients refractory to medical therapy or unfit for open surgery. Cannulation of the foramen ovale can be challenging due to anatomical variations. This article describes a hybrid technique combining fluoroscopy and electromagnetic neuronavigation to improve accuracy and safety. Preoperative CT planning allows individualized trajectory design, while intraoperative dual guidance minimizes multiple passes. Balloon compression is performed for 1–3 min to balance efficacy and complications. This minimally invasive procedure is effective, repeatable, and particularly suitable for elderly or comorbid patients, with a short learning curve for junior surgeons.

**Supplementary Information:**

The online version contains supplementary material available at 10.1007/s00701-026-06856-w.

## Relevant surgical anatomy

Lesioning of the trigeminal ganglion via a percutaneous approach is a well-established treatment option for trigeminal neuralgia. Main techniques include radiofrequency thermocoagulation, glycerol injection, and balloon compression [7]. The trigeminal nerve emerges from the pons and forms the Gasserian ganglion within Meckel’s cave. From this ganglion, the ophthalmic (V1), maxillary (V2), and mandibular (V3) divisions arise, with V2 and V3 most commonly affected [8, 10]. Cannulation of the foramen ovale (FO) provides direct access to the mandibular division and to the trigeminal ganglion.

The FO is an oval aperture in the posterior greater wing of the sphenoid bone, posterolateral to the foramen rotundum and anteromedial to the carotid canal. Its proximity to the cavernous sinus, internal carotid artery, and cranial nerves requires precise trajectory planning [3]. Variations in FO size, shape, or ligament ossification may complicate cannulation and increase procedural risk [3].

At the FO level, the mandibular nerve divides into anterior (mainly motor) and posterior (sensory) branches. Anatomical studies show the anterior branch lies anterior-lateral, while the sensory division is posteromedial [10]. This orientation is key for selective lesioning to optimize pain control while preserving motor function.

The classical Hartel approach uses surface landmarks and fluoroscopy to guide the needle to the FO [9]. However, fluoroscopy alone may be challenging in patients with anatomical distortions, often requiring multiple attempts. Neuronavigation systems, including frameless and electromagnetic guidance, enable real-time three-dimensional trajectory control and have demonstrated improved accuracy, fewer complications, and a shorter learning curve [2, 4, 5, 9].

A solid understanding of Meckel’s cave, the trigeminal ganglion, and FO anatomy is essential for safe and effective percutaneous rhizotomy. Combining fluoroscopy with neuronavigation increases precision and safety, particularly in complex anatomical scenarios.

## Description of the technique

**Fig. 1:**
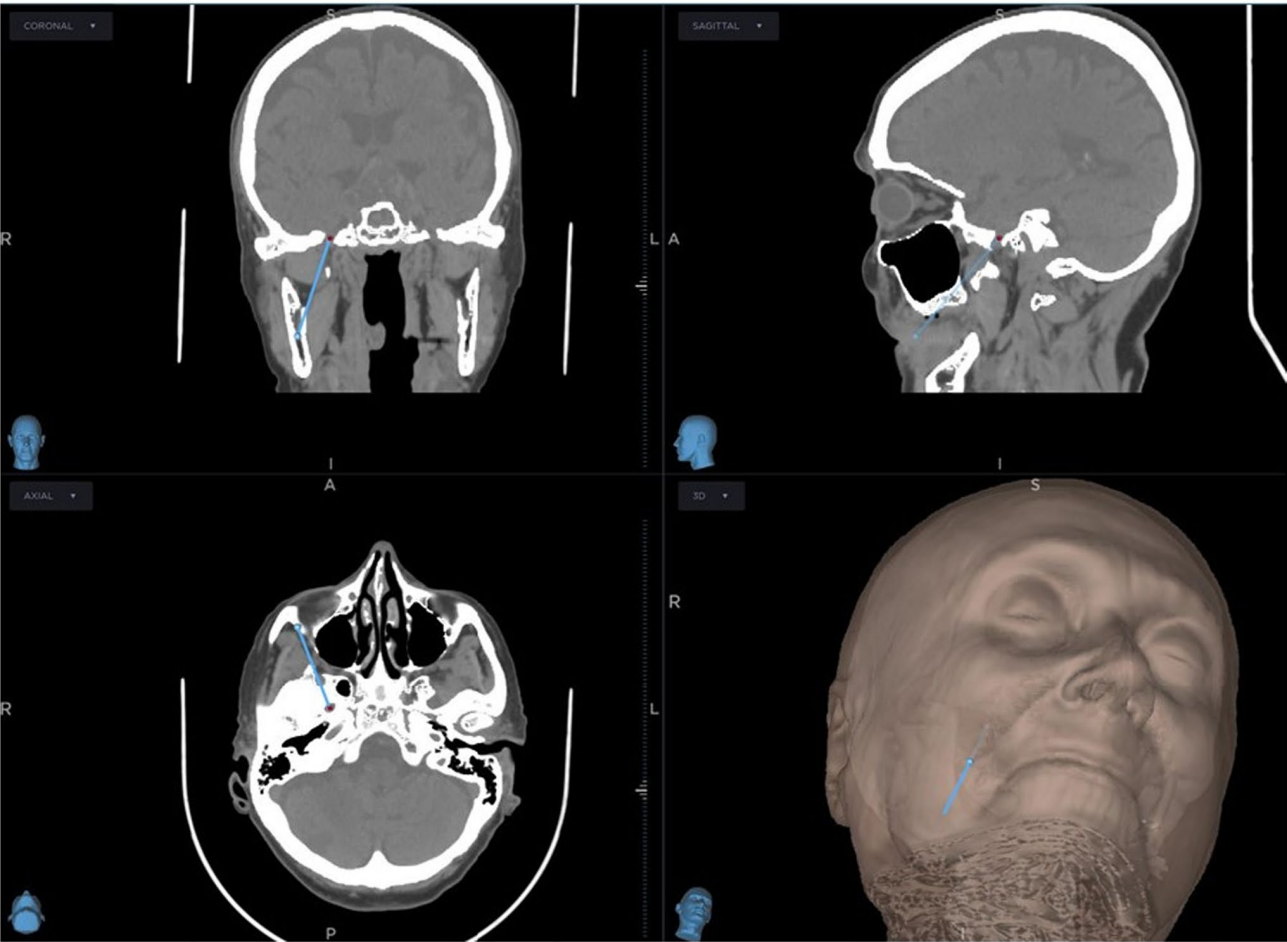
Preoperative neuronavigation planning for percutaneous balloon compression. Coronal, sagittal, and axial CT views demonstrate the planned needle trajectory (blue line) targeting the foramen ovale. A 3D surface reconstruction confirms the cutaneous entry point relative to facial landmarks, allowing trajectory optimization and avoidance of bony collision

On the day prior to surgery, patients undergo a thin-slice CT scan of the skull and facial skeleton for neuronavigation planning. The dataset is uploaded to the Medtronic StealthStation S8 system, where the needle trajectory is preoperatively designed to target the foramen ovale (Fig. 1). This planning step allows the surgeon to recognize anatomical variations and adapt the entry point when necessary, which may deviate from the classical Hartel approach.

The procedure is performed under general anesthesia (GA) with orotracheal intubation. This is essential because manipulation of the trigeminal ganglion may trigger a vagal reflex, potentially resulting in severe bradycardia or even asystole. The anesthesiologist must be prepared to promptly intervene, most often with intravenous atropine. Use of GA is also useful to reduce radiation exposure because of less patient movement.

**Fig. 2:**
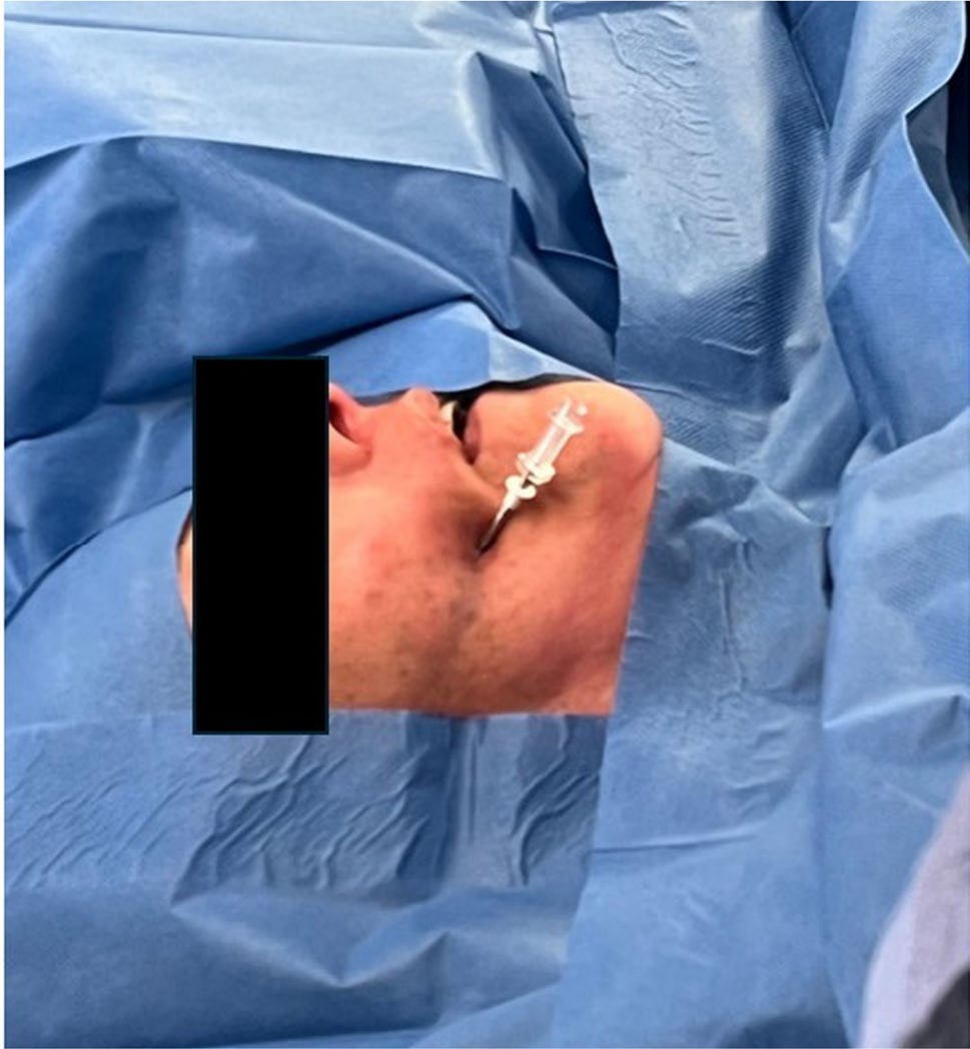
Intraoperative view showing the percutaneous entry point approximately 2.5 cm lateral to the oral commissure. The needle is secured and aligned with the preplanned trajectory toward the foramen ovale, as confirmed by neuronavigation and fluoroscopy. Proper positioning at this stage minimizes the need for trajectory adjustments and reduces the risk of mucosal injury

**Fig. 3:**
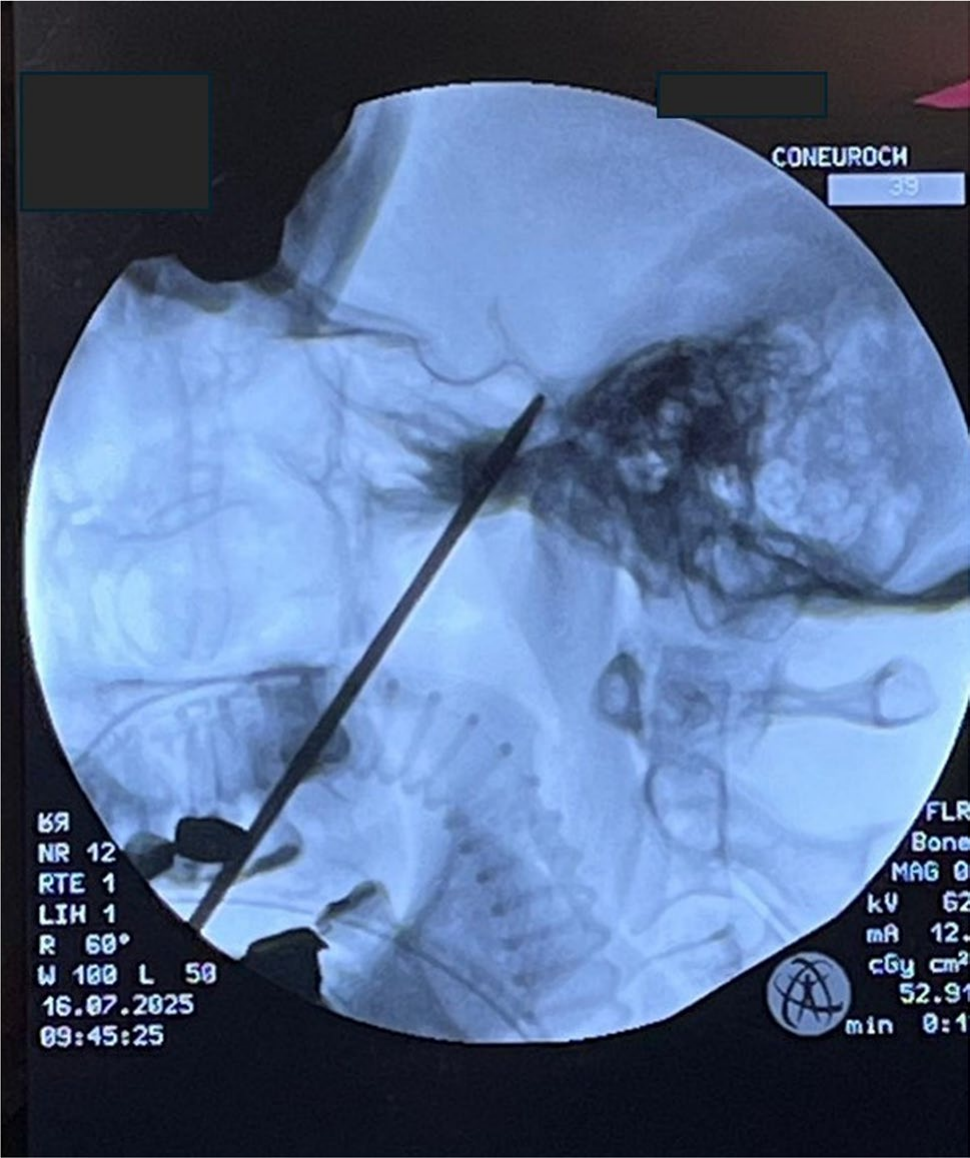
Intraoperative lateral fluoroscopic view showing the needle trajectory through the foramen ovale. Proper alignment is confirmed by the intersection of the needle tip with the petrous ridge–clivus junction, ensuring accurate cannulation of Meckel’s cave and optimal positioning for balloon insertion

**Fig. 4:**
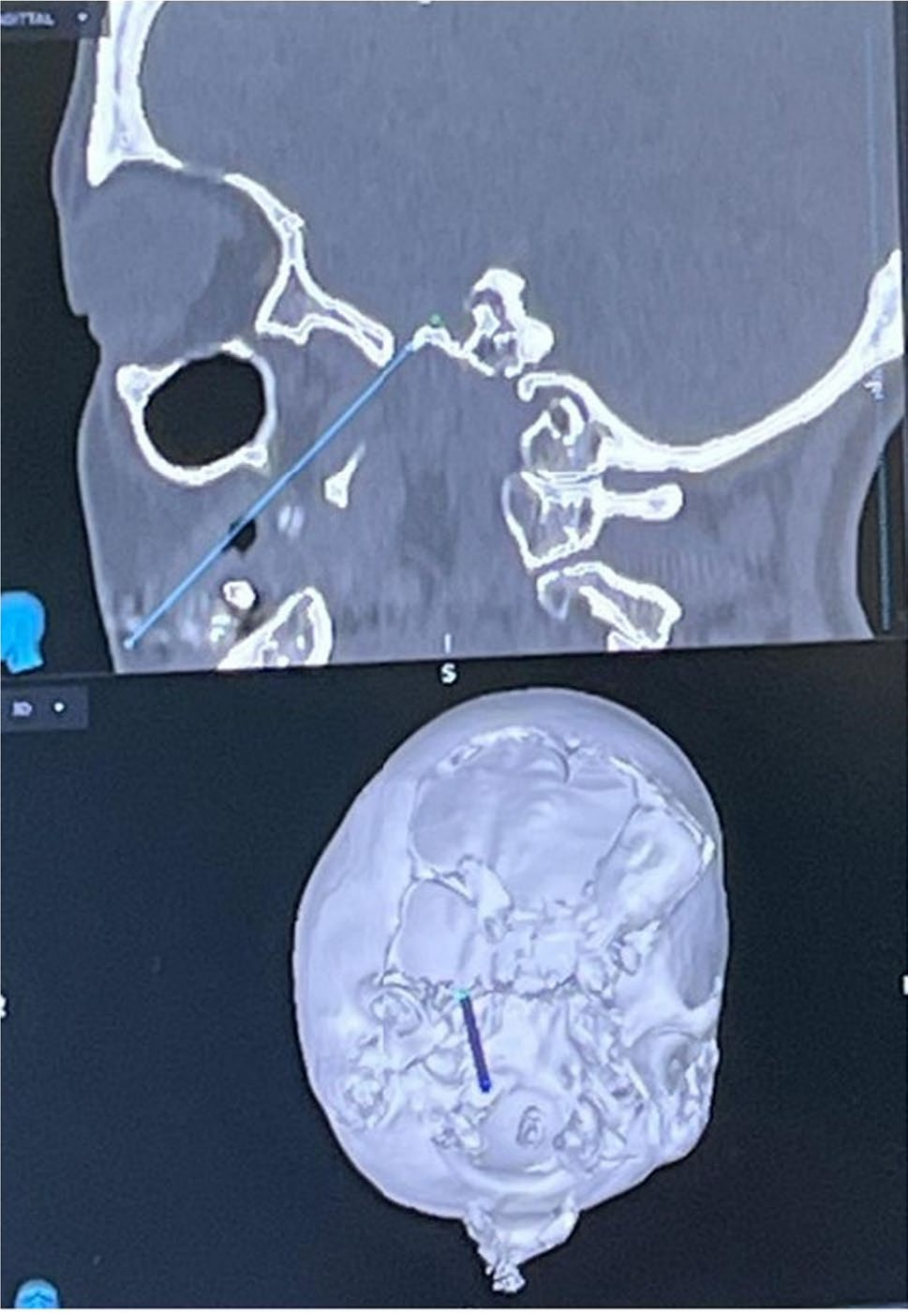
Intraoperative neuronavigation view showing real-time confirmation of the needle trajectory toward the foramen ovale. Sagittal CT reconstruction and 3D surface rendering allow precise alignment of the planned and actual paths, improving accuracy and reducing the need for multiple needle repositionings

The patient is positioned supine with the head placed on a U-shaped headrest and slightly extended to facilitate cannulation of the foramen ovale. Electromagnetic neuronavigation is used to confirm the skin entry site and the planned trajectory, while a C-arm fluoroscopy unit provides real-time confirmation of needle position during advancement and allows verification of the correct balloon inflation with contrast medium. The entry point is typically located approximately 2.5 cm lateral to the oral commissure on the affected side (Fig. 2). However, based on the preoperative planning performed with thin-slice CT and neuronavigation, the entry site may vary slightly from the classical Hartel point in order to account for individual anatomical variations of the foramen ovale and surrounding structures. To avoid injury of the oral mucosa, a finger of the non-dominant hand is placed within the oral cavity during initial needle advancement. The needle (KCA 11/15—10 access needle with double stylet, beveled and trocar tips, 11 balloon, balloon holder, 3 ml syringe with stopcock, and ergonomic wings – Tsunami Medical) is advanced under fluoroscopic control along the mid-pupillary plane toward the intersection of the petrous ridge and the clivus, verified on the lateral projection with alignment of the internal acoustic meatus (Fig. 3). Upon bony contact, the stylet is withdrawn and the Axiem electromagnetic sensor is introduced into the cannula to verify the accuracy of the trajectory compared with the preplanned navigation path (Fig. 4). Electromagnetic navigation was selected because it allows the use of a thin navigated stylet inserted inside the introducer, enabling real-time tracking of the cannula trajectory during advancement. Adjustments are made as needed before final advancement through the foramen ovale. A reflexive jaw jerk may be noted at this stage, and cerebrospinal fluid egress can occasionally occur but requires no specific intervention. After successful cannulation, a balloon catheter (4-French Fogarty) is introduced and inflated under fluoroscopic control with 0.75 ml of iopamidol contrast medium, producing the characteristic pear-shaped configuration. Balloon compression is maintained for approximately 1 to 3 min, balancing efficacy with the risk of hypoesthesia and masticatory weakness. At the end of the procedure, the cannula is removed and a medicated dressing is applied at the entry site.

## Indications

Percutaneous rhizotomy is an effective option for patients with trigeminal neuralgia (TN) resistant to medical therapy, especially after carbamazepine or oxcarbazepine failure [1]. In the absence of neurovascular conflict on MRI, it is preferred over microvascular decompression (MVD), particularly in elderly or comorbid patients unfit for open surgery [6]. It is also valuable in secondary TN due to multiple sclerosis, offering comparable efficacy across techniques (radiofrequency, glycerol rhizolysis, or balloon compression) and serving as a reliable palliative treatment [6].

## Limitations

The main limitation of this hybrid technique is the need for both electromagnetic neuronavigation and fluoroscopy, increasing costs and requiring personnel skilled in both systems. The learning curve can be initially demanding, but dual guidance provides a double-check mechanism, enhancing safety and confidence, especially for less experienced surgeons. Fluoroscopy also remains necessary during balloon compression to verify the correct inflation of the contrast-filled balloon and confirm its characteristic pear-shaped configuration.

Another limitation is the low level of evidence. Most studies on percutaneous balloon compression and radiofrequency rhizotomy are retrospective or small prospective cohorts. While navigation improves cannulation accuracy in complex cases, its superiority over single-modality guidance remains unproven, and randomized controlled trials are lacking [4, 6, 9, 10].

## How to avoid complications

Complications can be minimized by verifying the accuracy of the trajectory established during preoperative planning and ensuring proper calibration of the neuronavigation system. Intraoperatively, fluoroscopy must be carefully aligned, with the internal acoustic meatuses overlapping on the lateral projection, to confirm correct orientation. The anesthesiologist should be fully prepared for the occurrence of vagal reflex-induced bradycardia, with immediate administration of atropine if required. Compression time with the balloon should be strictly limited to a maximum of 3 min to reduce the risk of hypoesthesia and masticatory weakness.

## Specific information for the patient

Patients should be informed that this is a minimally invasive procedure, usually performed under short hospital stay. Immediate pain relief is expected in most cases, though transient facial numbness is common. Recurrence may occur over time, but the procedure can be repeated with similar efficacy. Potential risks include sensory disturbances, corneal hypoesthesia, masticatory weakness, and, rarely, anesthesia dolorosa.

## Key points summary

1. Hybrid fluoroscopy and neuronavigation improves accuracy of FO cannulation.
2. Preoperative CT planning allows tailored trajectories and avoids bony collision.
3. General anesthesia with atropine readiness is mandatory to manage vagal reflexes.
4. Entry point usually 2.5 cm lateral to the oral commissure but may vary with planning.
5. Dual-check mechanism provides greater safety and reduces multiple trajectories.
6. Balloon compression for 1–3 min balances efficacy and side effects.
7. Elderly and medically fragile patients are ideal candidates.
8. Evidence remains limited; no randomized trials available.
9. Hybrid approach may shorten the learning curve for junior surgeons.
10. The procedure is minimally invasive, repeatable, and generally well tolerated.

## Supplementary Information

Below is the link to the electronic supplementary material.

ESM 1 Supplementary Material 1 (MOV 465 MB)

## Data Availability

No datasets were generated or analysed during the current study.
